# Exploring How the Interplay Between Neighborhood Perceptions and Social Cohesion Relates to Self-Assessed Health: A Four-Way Decomposition with a 10-Year Follow Up

**DOI:** 10.1007/s11524-026-01083-1

**Published:** 2026-04-16

**Authors:** Vernon Cail, Joost Oude Groeniger, Mariëlle A. Beenackers, Frank J. van Lenthe

**Affiliations:** Department of Public Health, Erasmus MC University Medical Center Rotterdam, P.O. Box 2040, 3000 CA Rotterdam, South Holland The Netherlands

**Keywords:** Social environment, Social cohesion, Safety, Neighborhood disorder

## Abstract

The present study investigated the extent to which the relationship between perceptions of neighborhood unsafety and disorder and self-assessed health (SAH) is explained by mediation, interaction, or a combination of both through perceived neighborhood social cohesion. We analyzed data on 5650 respondents over a 10-year follow-up using the GLOBE study, a prospective cohort study in the Netherlands. Log-linear regression analyses were used to estimate the total effects of feeling unsafe and perceived neighborhood disorder with poor SAH. A four-way decomposition approach was used to decompose the total effects into four components: controlled direct effect (neither mediation nor interaction), pure indirect effect (mediation only), reference interaction effect (interaction only), and mediated interaction effect (both mediation and interaction). The results indicate that feeling unsafe had a positive estimated total effect on poor SAH (RR = 1.05; 95% CI, 1.01, 1.10). For perceived neighborhood disorder, the estimated total effect was smaller (RR = 1.01; 95% CI, 1.00, 1.04). Decomposition analysis indicated that the majority of the estimated effect of perceived unsafety was attributed to the controlled direct effect (ERR = 0.09; 95% CI, 0.05, 0.13), with no evidence of mediation or interaction through social cohesion. None of the estimated decomposition components were significant for neighborhood disorder. These findings suggest that perceived neighborhood disorder and unsafety and perceived neighborhood social cohesion appear to influence health through independent pathways, rather than through their interplay.

## Introduction

Over the past 20 years, there has been a growing body of research examining the relationship between neighborhood characteristics and health [1]. In this research, neighborhood conditions are often appraised by individuals’ perceptions of their neighborhood living environment [2]. Empirical evidence suggests that perceptions of neighborhood unsafety and disorder are linked to health outcomes [1]. Perceived neighborhood disorder refers to “visible cues indicating a lack of order and social control in the community” [3]. Such cues can be characterized as either physical disorder or social disorder. Neighborhoods with high physical disorder indicate the presence of litter, graffiti, and vandalism, whereas signs of social disorder include loitering, nuisances, and violence [3]. Previous research has shown that individuals who perceive their neighborhood as unsafe and having more disorder are more likely to have worse health outcomes than those who feel safe in their neighborhood and have less disorder [4–7]. However, the underlying pathways remain unclear.

A prominent framework for explaining the role of neighborhood disorder is the Broken Window Theory. This theory proposes that the presence of disorder may signal danger and threat, thereby hindering the development of social cohesion within communities [5, 8]. Since higher perceptions of neighborhood cohesion are associated with positive health outcomes [9], it may partly mediate the association between perceptions of neighborhood unsafety and disorder and health. Indeed, a 2022 longitudinal mediation analysis found that lower engagement in social activities partly explained the association between perceived crime and depressive symptoms in older European adults [10].

In addition to exploring potential mediation effects, it is important to consider the possibility of an interaction effect between perceptions of neighborhood unsafety and disorder and perceived neighborhood social cohesion. Communities with high levels of cohesion tend to have trusting relationships among residents, which promotes collective efficacy, informal social control, and reciprocity [11]. Such mechanisms might buffer the negative impact of unsafe feelings and perceived neighborhood disorder on health [12]. While a few studies have tested this hypothesis, findings are not consistent [7, 13, 14]. Moreover, extant research has only examined either mediation or interaction in isolation, without considering the potential co-occurrence of both mechanisms. Results from previous mediation analysis are therefore likely to be biased, since traditional mediation analysis assumes the absence of an interaction between exposure and mediator [15, 16].

This study draws on recent advances in counterfactual mediation analysis to assess both processes of mediation and interaction simultaneously, and to quantify the total effect between exposure and outcome into four components. Specifically, we aim to examine the extent to which the relationships between perceived neighborhood unsafety and neighborhood disorder and self-assessed health are explained by mediation, interaction, or a combination of both through perceived neighborhood social cohesion [17].

## Methods

### Data

**Fig. 1:**
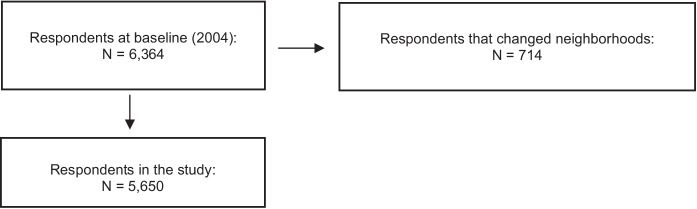
Flow chart of the respondents in the study

Data from this study come from the GLOBE Study, a prospective cohort study aimed at understanding socioeconomic inequalities in health in the Netherlands. Baseline measurements were taken in 1991 on a representative sample of 27,070 adults from the city of Eindhoven and surrounding villages [18]. A smaller subset was followed up in subsequent waves via postal surveys and oral interviews [18]. More details about the GLOBE study are available elsewhere [18]. This analysis included data from the follow-up surveys in 2004, 2011, and 2014. Participants that relocated to a different neighborhood during the follow-up period were excluded (see Fig. 1).

### Outcome

The outcome variable is self-assessed health (SAH) that was reported in 2014. Respondents were asked the single-item question on their perceived health: How would you rate your health in general? The five response categories ranged from poor, fair, good, very good, and excellent. The variable was dichotomized into poor health (poor and fair) and good health (good, very good, and excellent), with good health serving as the reference.

### Exposures

Perceived neighborhood unsafety was assessed in 2004 using three items. Respondents were asked the extent they agree with three statements about the safety of their neighborhood: (1) “In this neighborhood, I am sometimes afraid to walk the streets alone at night,” (2) “In this neighborhood, I am sometimes afraid to walk the streets alone in the daytime,” and (3) “In this neighborhood, I am sometimes afraid to stay home alone at night.” Response categories were “disagree,” “neither agree nor disagree,” and “agree,” ranging from 0 to 2, respectively. Following the work of Kamphuis et al. [19], a composite score was constructed by summing the three items, with a high score indicating more unsafe feelings.

Perceived neighborhood disorder was measured with eleven items, which asked respondents how often they think certain bad incidents occur in their neighborhood. Bad incidents included litter, vandalism, graffiti, youth nuisance, bicycle thefts, breaking into cars, breaking into houses, drunkenness on the street, harassment on the street, threats, and violent crimes. Response categories included almost never (1), sometimes (2), and often (3). Following the previous research [20, 21], we summed the 11 items to construct a composite score indicating an overall assessment of local problems, with the higher score indicating greater problems in the neighborhood.

### Mediator

Perceived neighborhood social cohesion was assessed in the 2011 follow-up survey using a four-item scale based on two widely used instruments [11, 22, 23]. Respondents were asked to what extent they agree or disagree with the following questions about their neighborhood: (a) People in this neighborhood get along with each other pleasantly, (b) People in this neighborhood are willing to help each other, (c) I often feel alone in this neighborhood, and (d) I would move out of this neighborhood if I get the chance. The first two items capture perceived collective efficacy, whereas the last two items measure a sense of belonging [23]. Response categories were “strongly agree,” “agree,” “neither agree nor disagree,” “do not agree,” and “strongly do not agree,” ranging from 1 to 5. The four items were summed and reverse coded so that higher values indicate less social cohesion.

### Covariates

This analysis adjusted for the following individual sociodemographic characteristics from 2004: gender (male, female), age (years), living arrangement (living with a partner, living without a partner), educational level according to the International Standard Classification of Education (low = ISCED 0–2, middle = ISCED 3–4, high = ISCED 5–8), household income (< €1200, €1200–1800, €1800–2600, > €2600), employment status (employed, unemployed, retired), and baseline SAH (good health, poor health).

### Data analysis

Descriptive statistics were used to describe the baseline sociodemographic characteristics and neighborhood perceptions of the study population in 2004, social cohesion measurements in 2011, and SAH in 2014.

Log-linear regression models were used to assess the overall relationships of perceptions of unsafe feelings and perceived neighborhood disorder and poor SAH, and to assess the relationship between perceived neighborhood social cohesion and poor SAH. Estimates were reported as relative risks with their 95% confidence intervals. Linear regression models were used to assess the relationships of the two exposures with perceived neighborhood social cohesion, with estimates reported as beta coefficients and 95% confidence intervals. Missing values for exposures, mediator, outcome, and baseline characteristics were handled by using the multivariate imputation by chained equations package in *R* (*M* = 20) [24].

**Fig. 2:**
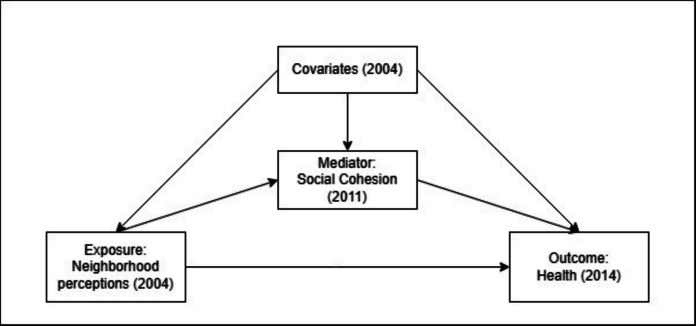
Directed acyclic graph for the hypothesized effect of neighborhood perceptions on self-assessed health, mediated by social cohesion, and adjusted for baseline covariates: gender, age, living arrangement, educational level, household income, employment status, and baseline self-assessed health

A four-way decomposition framework was used to estimate the mediating and interaction effects of perceived neighborhood social cohesion on the relationships between perceptions of unsafe feelings and perceptions of neighborhood disorder and poor SAH. This approach decomposes the total effect into four components: a controlled direct effect, a reference interaction, a mediated interaction, and a pure indirect effect [17]. A detailed description of each component’s definition based on the counterfactual framework and their interpretation in the context of the current study is presented in Table 1. In addition to the consistency and positivity assumptions, the four-way decomposition requires the assumptions of no unmeasured confounding of the exposure–outcome, exposure–mediator, and mediator–outcome relationships, and that there are no mediator–outcome confounders that are themselves affected by the exposure [17]. These assumptions are encoded in the Directed Acyclic Graph depicted in Fig. 2 that underlies our analytical approach. The four-way decomposition analysis was conducted in the CMAverse package in R, which applied multiple imputation internally [25]. The reference level of the mediator was set to its median (i.e., 9) and 95% confidence intervals were obtained by normal distribution approximation. Estimates were expressed as excess relative risk (ERR), which was calculated as the relative risk minus one (ERR = RR − 1). All models in this study were adjusted for baseline sociodemographic and baseline health status. All analyses were performed in R 4.2.2 [26].

**Table 1:** Definitions of the four-way decomposition as applied to the current study

Causal estimate	Notation [17]	Interpretation
Controlled direct effect (CDE)	*Y*_10_ − *Y*_00_	The effect of perceived neighborhood disorder/unsafety on SAH that is neither mediated through nor modified by perceived neighborhood social cohesion. In other words, perceived neighborhood disorder/unsafety affects SAH through pathways completely independent of perceived neighborhood social cohesion
Pure indirect effect (PIE)	(*Y*_01_ − *Y*_00_)(*M*_1_ − *M*_0_)	The effect of perceived neighborhood disorder/unsafety on SAH that operates through perceived neighborhood social cohesion as a mediator, in the absence of interaction. This means perceived neighborhood disorder/unsafety improves health solely because it increases perceived neighborhood social cohesion, which in turn improves SAH
Reference interaction (INTref)	(*Y*_11_ − *Y*_10_ − *Y*_01_ + *Y*_00_)(*M*_0_)	The effect attributable to interaction between perceived neighborhood disorder/unsafety and perceived neighborhood social cohesion, but not through mediation. This means the effect of perceived neighborhood disorder/unsafety on SAH differs depending on the level of perceived neighborhood social cohesion, but this is not because perceived neighborhood disorder/unsafety caused perceived neighborhood social cohesion
Mediated interaction (INTmed)	(*Y*_11_ − *Y*_10_ − *Y*_01_ + *Y*_00_)(*M*_1_ − *M*_0_)	The effect of perceived neighborhood disorder/unsafety on SAH attributable to both mediation by and interaction with perceived neighborhood social cohesion. This means perceived neighborhood disorder/unsafety affects perceived neighborhood social cohesion, and perceived neighborhood social cohesion in turn modifies the effect of perceived neighborhood disorder/unsafety on health

## Results

Of the 6463 respondents who participated at baseline, 714 were excluded due to moving to a different neighborhood during the study period, resulting in 5650 participants for the analyses. Table 2 presents a summary of the study population. The average score for unsafe perceptions was 0.8 out of a possible score of 6, while the average score for perceptions of neighborhood disorder was 14.7 out of a possible score of 33. Approximately 9% of the study population reported poor SAH. The average age of the study population was 56.

**Table 2:** Descriptive statistics of the study population

	*N* (%) or mean (SD)	Median (min, max)
Sample size	5650 (100.0%)	
*Covariates*		
Gender		
Male	2631 (46.6%)	
Female	3019 (53.4%)	
Age	56.6 (15.6)	59.0 (23, 90)
Missing	66 (1.2%)	
Living arrangements		
Living without a partner	1145 (20.3%)	
Living with a partner	4194 (74.2%)	
Missing	311 (5.5%)	
Education		
Low	2555 (45.2%)	
Middle	1192 (21.1%)	
High	1500 (26.5%)	
Missing	403 (7.1%)	
Household income		
< 1200	781 (13.8%)	
1200–1800	1229 (21.8%)	
1800–2600	1332 (23.6%)	
> 2600	1431 (25.3%)	
Missing	877 (15.5%)	
Employment status		
Employed	1216 (44.6%)	
Retired	759 (27.9%)	
Unemployed	562 (20.6%)	
Missing	187 (6.9%)	
Baseline self-assessed health		
Good health	4424 (78.3%)	
Poor health	1062 (18.8%)	
Missing	164 (2.9%)	
*Exposures*		
Perceived unsafety	0.8 (1.3)	0 (0, 6)
Missing	142 (2.5%)	
Perceived disorder	14.7 (3.6)	14 (11, 33)
Missing	352 (6.2%)	
*Mediator*		
Social cohesion	8.8 (2.5)	8 (5, 20)
Missing	2838 (50.2%)	
*Outcome*		
Self-assessed health		
Good health	2107 (37.3%)	
Poor health	490 (8.7%)	
Missing	3053 (54.0%)	

The estimated effects of perceived unsafety and neighborhood disorder on reporting poor SAH are presented in Table 3. A one unit increase in feeling unsafe was associated with a 5% higher probability of reporting poor SAH while adjusting for baseline characteristics (RR = 1.05; 95% CI, 1.01, 1.10), while a one unit increase in perceptions of neighborhood disorder was associated with a 1% increase in the probability of reporting poor SAH (RR = 1.01; 95% CI, 1.00, 1.04).

**Table 3:** Estimated effects of perceptions of feeling unsafe and perceptions of neighborhood disorder on reporting poor self-assessed health

	Perceived unsafety		Perceived disorder	
	Model 1	Model 2	Model 1	Model 2
	RR (95%CI)	RR (95%CI)	RR (95%CI)	RR (95%CI)
Perceived unsafety	1.05 (1.01, 1.10)	1.04 (0.99, 1.09)		
Perceived disorder			1.01 (1.00, 1.04)	1.01 (1.00, 1.03)
Social cohesion*		1.04 (1.01, 1.08)		1.04 (1.01, 1.08)
Gender				
Male	Ref	Ref	Ref	Ref
Female	0.99 (0.86, 1.14)	1.01 (0.87, 1.16)	1.02 (0.89, 1.19)	1.03 (0.89, 1.19)
Age	1.01 (1.00, 1.02)	1.01 (1.00, 1.02)	1.01 (1.00, 1.02)	1.01 (1.00, 1.02)
Living arrangement				
Living alone	Ref	Ref	Ref	Ref
Living with partner	0.85 (0.72, 1.01)	0.87 (0.74, 1.04)	0.86 (0.72, 1.02)	0.88 (0.74, 1.05)
Education level				
Low	Ref	Ref	Ref	Ref
Middle	0.74 (0.58, 0.95)	0.73 (0.57, 0.94)	0.73 (0.57, 0.94)	0.73 (0.57, 0.93)
High	0.64 (0.50, 0.83)	0.64 (0.50, 0.82)	0.64 (0.50, 0.82)	0.63 (0.49, 0.81)
Income				
< 1200	Ref	Ref	Ref	Ref
1200–1800	1.05 (0.84, 1.30)	1.06 (0.85, 1.31)	1.04 (0.84, 1.29)	1.05 (0.85, 1.30)
1800–2600	0.95 (0.74, 1.22)	0.98 (0.76, 1.26)	0.95 (0.73, 1.22)	0.97 (0.75, 1.25)
> 2600	0.87 (0.64, 1.19)	0.90 (0.66, 1.24)	0.87 (0.64, 1.19)	0.90 (0.66, 1.23)
Employment status				
Employed	Ref	Ref	Ref	Ref
Retired	1.17 (0.88, 1.57)	1.18 (0.88, 1.57)	1.19 (0.89, 1.58)	1.19 (0.89, 1.58)
Unemployed	1.13 (0.90, 1.43)	1.12 (0.89, 1.42)	1.13 (0.90, 1.42)	1.12 (0.89, 1.42)
Baseline health				
Good health	Ref	Ref	Ref	Ref
Poor health	3.73 (3.16, 4.41)	3.61 (3.04, 4.28)	3.76 (3.18, 4.45)	3.63 (3.06, 4.31)

Model 1 includes baseline covariates (measured in 2004). Model 2 includes baseline covariates (measured in 2004) and social cohesion (measured in 2011). Social cohesion was reverse coded so that a high value means less cohesion*

The estimated effects of perceived neighborhood social cohesion on reporting poor SAH are also presented in Table 3. The results indicated that perceiving less social cohesion was associated with a higher probability of reporting poor SAH, controlling for either perceptions of unsafety or perceived neighborhood disorder (RR = 1.04; 95% CI, 1.01, 1.08).

Table 4 presents the estimated effects of perceived unsafety and neighborhood disorder on (reverse coded) perceived neighborhood social cohesion. We observed that a higher perception of feeling unsafe (*β* = 0.31; 95% CI, 0.23, 0.40) and more perceived neighborhood disorder (*β* = 0.11; 95% CI, 0.08, 0.14) were associated with less perceived neighborhood social cohesion.

**Table 4:** Estimated effects of perceptions of feeling unsafe and perceptions of neighborhood disorder on perceived neighborhood social cohesion

	Perceived unsafety	Perceived disorder
	*β* (95%CI)	*β* (95%CI)
Perceived unsafety	0.31 (0.23, 0.40)	
Perceived disorder		0.11 (0.08, 0.14)
Gender		
Male	Ref	Ref
Female	−0.42 (−0.62, −0.23)	−0.26 (−0.46, −0.06)
Age	−0.02 (−0.03, −0.01)	−0.02 (−0.03, −0.01)
Living arrangement		
Living alone	Ref	Ref
Living with partner	−0.60 (−0.85, −0.34)	−0.56 (−0.82, −0.31)
Education level		
Low	Ref	Ref
Middle	0.16 (−0.13, 0.45)	0.10 (−0.19, 0.39)
High	0.22 (−0.03, 0.47)	0.16 (−0.09, 0.41)
Income		
< 1200	Ref	Ref
1200–1800	−0.26 (−0.65, 0.13)	−0.33 (−0.71, 0.05)
1800–2600	−0.70 (−1.06, −0.33)	−0.73 (−1.10, −0.37)
> 2600	−0.84 (−1.21, −0.47)	−0.85 (−1.23, 0.48)
Employment status		
Employed	Ref	Ref
Retired	0.15 (−0.22, 0.51)	0.19 (−0.17, 0.56)
Unemployed	0.29 (0.02, 0.57)	0.29 (0.02, 0.56)
Baseline health		
Good health	Ref	Ref
Poor health	0.90 (0.62, 1.19)	0.14 (0.66, 1.22)

The results of the four-way decomposition analysis are presented in Table 5. There was a positive direct effect estimate between perceptions of unsafe feelings and reporting poor SAH (CDE ERR = 0.09; 95% CI, 0.05, 0.13). The INTref (ERR = −0.00; 95% CI, −0.06, 0.05), INTmed (ERR = 0.00; 95% CI, −0.01, 0.01), and PIE (ERR = 0.01; 95% CI, −0.00, 0.03) were close to null. Results for perceptions of neighborhood disorder were similar, except that the positive direct effect estimate was much smaller, and confidence intervals included the null (CDE ERR = 0.03; 95% CI, −0.06, 0.11).

**Table 5:** Four-way decomposition of the estimated effects of perceptions of feeling unsafe and perceptions of neighborhood disorder mediated by perceived neighborhood social cohesion

	Perceived unsafety	Neighborhood disorder
	ERR (95% CI)	ERR (95%CI)
CDE	0.09 (0.05, 0.13)	0.03 (−0.06, 0.11)
INTref	−0.00 (−0.06, 0.05)	0.00 (−0.08, 0.08)
INTmed	0.00 (−0.01, 0.01)	−0.00 (−0.00, 0.00)
PIE	0.01 (−0.00, 0.03)	0.01 (−0.00, 0.03)

Models are adjusted for baseline covariates of gender, age, living arrangement, educational level, household income, employment status, and self-assessed health

*ERR* excess relative risk, *CDE* controlled direct effect, *INTref* reference interaction, *INTmed* mediated interaction, *PIE* pure indirect effect

## Discussion

This study found that perceived neighborhood unsafety was associated with a higher probability of poor SAH, independently of perceived neighborhood social cohesion. Although perceived neighborhood social cohesion was also positively associated with SAH, the four-way decomposition analysis revealed that the estimated total effect of perceived neighborhood unsafety on SAH was attributable entirely to its pure direct effect, indicating that social cohesion neither mediated nor interacted with this relationship. The relationship between perceived neighborhood disorder and SAH was much weaker.

This study contributes to the literature in a number of ways. First, a key strength of this study is its longitudinal design, which allowed us to examine the pathways linking perceptions of unsafety and neighborhood disorder, social cohesion, and health outcomes while establishing temporal precedence. Second, we applied a four-way decomposition framework, which, to our knowledge, has not been used in this context to disentangle potential mediation and interaction effects of social cohesion on the relationships between perceptions of unsafe feeling, neighborhood order, and SAH.

However, this study also has several limitations to consider. An important limitation is the long interval between measurements: the exposure was measured in 2004, the mediator in 2011, and the outcome in 2014. While this study design establishes temporal precedence, the long gap limits the ability to capture causal dynamics between perceptions of neighborhood unsafety/disorder and social cohesion. As a result, measurement error in the mediator is possible, which can attenuate estimates of indirect effects and may partially explain the limited evidence for mediation [15]. Additionally, the reliance on self-reported data may introduce same-source bias and additional measurement errors. This could result in inflating the relationship between these variables [27]. Attrition bias due to a large loss to follow-up was another concern, which we try to mitigate using multiple imputation to account for missing data. However, this approach is only valid under the assumption that missingness was random given the observed data included in our imputation model. The four-decomposition analysis is based on the assumptions of conditional exchangeability [17]. While we attempted to address this assumption by adjusting for baseline sociodemographic characteristics and baseline health status, unmeasured confounding, including potential time-varying confounders influenced by prior exposure, may still exist.

Our findings indicate that the relationship between perceived neighborhood unsafety and health is consistent with previous research, providing further evidence that perceiving the neighborhood as safe is beneficial to individual health [7, 28]. This relationship seemed stronger for perceived unsafety than for perceived disorder, which aligns with previous research showing an inconsistent relationship between perceived neighborhood disorder and health [5, 29]. One possible explanation for the stronger health impact of safety feelings is that feeling unsafe may trigger stronger stress responses than observing neighborhood disorder. Residents may not always interpret signs of neighborhood disorder as a personal threat, particularly if they have become accustomed to it. These findings suggest that policies aiming to improve population health should prioritize enhancing residents’ sense of safety, rather than focusing solely on reducing visible signs of disorder.

The results from the four-way decomposition analysis indicate that the majority of the relationship between perceived unsafety and SAH was attributable to the direct pathway. Furthermore, we found no evidence that perceived neighborhood social cohesion mediated or interacted with this relationship. This suggests that social cohesion may not be a primary mechanism linking neighborhood perceptions to health. Few studies have investigated the mediating role of social cohesion in the association between perceived (un)safety and health, limiting opportunities for comparison. This gap in the literature may reflect the difficulty of disentangling perceived safety from social cohesion, as these constructs show some conceptual and empirical overlap [30, 31]. Future research could benefit from adopting a pluralistic approach, combining qualitative and quantitative methods to better capture the complex interplay of different neighborhood characteristics in shaping health outcomes.

In conclusion, this study found that perceived neighborhood safety was associated with better SAH independently of perceived neighborhood social cohesion. Although social cohesion was also associated with better SAH, it seems to neither carry nor modify the health benefits of higher perceived neighborhood safety. These findings suggest that neighborhood safety and social cohesion represent distinct and independent pathways to better health and that the interventions targeting either dimension may yield separate health benefits without relying on or amplifying the other.

## Data Availability

The data underlying this article is available upon reasonable request and with specific restrictions. Access can be requested from the corresponding author.
